# Psychotic-Like Experiences and Their Cognitive Appraisal Under Short-Term Sensory Deprivation

**DOI:** 10.3389/fpsyt.2014.00106

**Published:** 2014-08-15

**Authors:** Christina Daniel, Anna Lovatt, Oliver John Mason

**Affiliations:** ^1^Research Department of Clinical, Educational and Health Psychology, University College London, London, UK; ^2^Cheshire and Wirral Partnership NHS Foundation Trust, Chester, UK

**Keywords:** sensory deprivation, psychosis proneness, appraisals, anomalous body experiences, hallucinations

## Abstract

**Aims:** This study aimed to establish and compare the effects of brief sensory deprivation on individuals differing in trait hallucination proneness.

**Method:** Eighteen participants selected for high hallucination proneness were compared against 18 participants rating low on this trait. The presence of psychotic-like experiences (PLEs), and participants’ cognitive appraisals of these, was evaluated in three different settings: at baseline, in a “secluded office” environment, and in light-and-sound sensory deprivation.

**Results:** Psychotic-like experiences were experienced significantly more often in sensory deprivation for both groups. In particular, both experienced slight increases in perceptual distortions and anhedonia in seclusion, and these increased further during sensory deprivation. Highly hallucination prone individuals showed a significantly greater increase in perceptual distortions in sensory deprivation than did non-prone individuals suggesting a state-trait interaction. Their appraisals of these anomalous experiences were compared to both clinical and non-clinical individuals experiencing psychotic symptoms in everyday life.

**Conclusion:** Short-term sensory deprivation is a potentially useful paradigm to model psychotic experiences, as it is a non-pharmacological tool for temporarily inducing psychotic-like states and is entirely safe at short duration. Experiences occur more frequently, though not exclusively, in those at putative risk of a psychotic disorder. The appraisals of anomalous experiences arising are largely consistent with previous observations of non-clinical individuals though importantly lacked the general positivity of the latter.

## Introduction

Since around 2000 high risk research has increasingly investigated how psychotic-like experiences (PLEs) may be part of the risk trajectory for psychosis (1, 2). However, there is a long history of experimental paradigms attempting to induce such experiences in healthy individuals, much of it taking place back in the 1950s and 1960s. Many of these studies employed sensory deprivation of various kinds as the method for inducing anomalous experiences [e.g., Ref. (3)]. The findings were inconsistent, possibly due to an inadequate recognition of the complexity of the variables that enter into the situation of sensory restriction (4). Prolonged periods of deprivation were found to produce a range of psychotic-like phenomena in many, if not all participants. However, experiences at shorter durations varied depending on the nature of the deprivation, and the characteristics of the participants involved. Other studies (5, 6) concluded that PLEs occur in highly suggestible individuals who have a tendency to mistake “imaginary” events as veridical. Researchers lost interest in the field of inducing anomalous experiences, many dismissing the phenomena as more akin to fantasy or acts of imagination and not a true parallel of the hallucinations and other positive symptoms seen in psychosis.

While PLEs are interesting in their own right, we would propose that the theoretical construct of schizotypy conceived as a continuous dimension of risk is an important theoretical perspective from which to interpret individual differences in PLEs. Furthermore, the study of individuals with schizotypal characteristics has the advantage that results are not confounded by the contribution of variables such as hospitalization, medication effects, illness duration, and cognitive deficits. Within the theoretical framework of the continuum hypothesis, the study of a subclinical sample can lead the way toward understanding the perceptual–cognitive mechanisms underlying anomalous experiences both in and outside of psychosis.

With the potential utility for studying PLEs in the normal population now clearly re-established as part of the psychosis continuum (1) and schizotypy rubrics, researchers have taken a fresh look at methods to experimentally induce such experiences, with perhaps the most widely studied relating to the induction of auditory–verbal hallucinations. Many of these methods have been informed by recent theoretical accounts of “voice hearing” such as increased impact of top-down processing (7, 8), reality discrimination failure (9), and increased sensitivity to internally generated percepts (10). Overall, several studies suggest a tendency to erroneously allocate an external source to internally generated stimuli may underlie hallucination proneness in schizophrenia [for review see Ref. (11)]. These findings support a tentative hypothesis that in sensory deprivation, where external events are absent or minimal, highly schizotypal, or hallucination prone individuals are more likely to erroneously process inner thoughts as being external events, and hence experience PLEs. A more sophisticated Bayesian framework (12, 13) suggests that psychotic symptoms arise from a disturbance in error-dependent updating of inferences and beliefs about the world. Corlett et al. (12), in particular, point out how several psychotomimetic drugs may come to have their effects by temporarily disturbing this cognitive process. They go on to discuss how sensory deprivation “may share phenomenological and biological similarities with the serotonergic hallucinogens, both occur when the top-down system imposes structure on noisy, unpredictable bottom-up signals” (p. 524).

In the modern era, several studies have attempted to use a sensory deprivation paradigm to induce PLEs in the normal population (14–18). Using more modern techniques, all studies were successful in inducing hallucinations of varying complexity in many of the participants. What is more, PLEs have been shown to be successfully induced using such methods with as little as 15 min exposure to deprivation of sight and sound (17). Mason and Brady (17) used an anechoic chamber (an environment of total light-and-sound deprivation) to induce PLEs (perceptual disturbances, paranoia, and anhedonia) particularly in those prone to hallucinatory experiences. This pilot study had a number of methodological limitations, not least its sample size of only 19 in total. The study was also criticized for the fact that the procedure included a “panic” button (19) on the basis that a previous study (20) showed the group with just such a button reported many more perceptual aberrations, and cognitive and emotional disturbances, including heightened anxiety. Bell (19) also suggested that the increased PLEs in the high hallucination prone group might be accounted for by differential anxiety levels between high and low-prone groups. This is a serious potential confound, it has been demonstrated that hallucination proneness is linked to trait anxiety (21), and in individuals with psychosis, acute anxiety is clearly linked to an increase in hallucinatory experiences (22). Therefore, it is feasible that an increase in anxiety brought about by sensory deprivation acts to mediate the relationship between PLEs and hallucination proneness. Anxiety was not measured in the original study and this omission was a major limitation. Assessment at baseline was also an area for technical improvement. The pilot study assessed this prior to entering the anechoic chamber when preparatory anxiety may have been influential. The current study utilized a further “secluded office” environment condition as a potentially better matched control condition than standard “baseline.”

Cognitive models of psychotic symptoms (23) place central emphasis on how anomalous experiences are appraised. Among the range of appraisals, those that are externalizing and personalizing are thought to play a significant role in determining the transition to clinical psychosis and so are considered of particular significance. Garety et al. (23) also suggested that some people who have anomalous experiences may reject external attributions and so be protected in some way from developing full-blown psychotic symptoms such as delusions. Appraisals and the continuum of psychotic experiences have been studied in depth by Brett et al. (24). Their measure, the appraisals of anomalous experiences interview (AANEX), assesses anomalous experiences and individuals’ responses to them, including their appraisals. Individuals with schizophrenia spectrum disorders appraised their experiences as more negative, more dangerous, more likely to be external and personally caused, and made more paranoid/conspiracy interpretations than non-diagnosed controls. Several subsequent studies (25, 26) have further elucidated, which AANEX appraisals distinguish clinical from “at risk” and healthy samples, and which predict greater distress. Assessment of appraisal of PLEs during sensory deprivation has not been described and is novel to this study.

### Aims and hypotheses

The current study aimed to replicate the effects of brief sensory deprivation using an anechoic chamber in larger groups of low and high hallucination prone individuals. A one-way microphone was also used to monitor participants rather than using a panic-button in an attempt to reduce potential demand characteristics. In addition, state and trait anxiety were measured as potential confounds/covariates. A further aim was to characterize the cognitive appraisals of PLEs arising in sensory deprivation using the AANEX and compare these to existing data.

It was hypothesized that:

1. The high schizotypy group would exhibit a greater degree of PLEs than the low group under normal baseline conditions. This helps further validate the measure of PLEs.
2. Both high and low hallucination prone groups would experience a significant increase in psychotic-like symptoms from baseline in sensory deprivation.
3. The increase in psychotic-like symptoms in sensory deprivation would be significantly greater for the high schizotypy group than the low schizotypy group.
4. The above effects would remain after controlling for any state/trait anxiety differences between the groups.

## Materials and Methods

### Participants

Participants between the ages of 18 and 65 years were recruited via a university psychology department website that advertises to both students and the general public. Exclusion criteria included a history of a major psychiatric or neurological disorder, or recreational or psychotropic drug use in the past 3 months. An advert was placed inviting participants to complete an online version of the revised hallucinations scale [RHS: (27)]. Three hundred seventeen participants from a wide range of ethnic backgrounds returned data. From this sample, 76 low scorers and 39 high scorers were invited to participate as these conformed to the upper and lower 20th percentiles, according to questionnaire norms. Of these, 18 low scorers (7 males, 11 females, mean age = 25.39 years, SD = 6.09, mean score = 26.22, SD = 1.77) and 18 high scorers (4 males, 14 females, mean age = 24.94 years, SD = 3.95, mean score = 54.94, SD = 5.25) gave informed consent, consistent with university ethical procedures.

### Power analysis

Very little is known about the effects of sensory deprivation on people who rate highly for hallucination proneness, and so it was challenging to accurately estimate effect sizes from existing literature. The most similar study to date (17) reported large effect sizes for increases in perceptual distortions (partial η^2^ = 0.56) and anhedonia (partial η^2^ = 0.58) measured using the psychotomimetic states inventory (PSI) (28) immediately after 15 min of sensory deprivation. The power calculation for the current study was based on the smaller of these effect sizes – partial η^2^ = 0.56. This is a conservative estimate for current purposes since participants in the current study spent a longer length of time in sensory deprivation (25 min) presumably providing greater opportunity for perceptual distortions to arise. Power calculations suggested that a minimum total sample of 18 per group would provide statistical power for a between-within participants repeated measures ANOVA design that exceeded 80% (β = 0.80), with α = 0.05.

### Measures

#### Revised hallucinations scale

This is a 24-item questionnaire based on the Launay–Slade hallucination scale (29) measuring a predisposition to experience hallucinations. It uses a revised scoring method, which allows participants to respond on a 4-point scale (1 = never to 4 = almost always). The scale has been shown to have good reliability and predictive validity, and moderately stable internal consistency over a period of 4–6 weeks (27).

#### Psychotomimetic states inventory

This is a 48-item questionnaire measuring psychosis-like experiences. Items are rated on a 4-point scale (from 0 = never to 3 = strongly), with some items being reverse scored (28). The PSI has sub-scales of delusory thinking, perceptual distortions, cognitive disorganization, anhedonia, mania, and paranoia. Originally developed for use in drug studies, it has produced meaningful results in a previous preliminary study of sensory deprivation (17).

#### State-trait anxiety inventory

A pair of two 20-item questionnaires that measure the temporary condition of state anxiety, and the more longstanding quality of trait anxiety. Items are rated of a 4-point scale. The state-trait anxiety inventory (STAI) has been shown to have good construct validity with multiple other assessment tools. It has also been shown to have good test-retest reliability [0.54 correlation for state, and 0.86 correlation for trait anxiety (30)].

#### Appraisals of anomalous experiences interview

A multidimensional measure of psychological responses to anomalies associated with psychosis (24). The first section (the AANEX inventory) includes items reflecting schneiderian first-rank symptoms and anomalies of perception, cognition, affect, and “individuation” (sense of distinction between self and others), as well as some “paranormal” experiences. The inventory generates two sets of scores: “lifetime” (not used in this study) and “state.” For state scores, items are rated between 0 and 2 (absent, marginal, and present). The present study assessed whether a particular experience could be used to generate a state score.

The second section (the AANEX-CAR) is a structured interview that assesses appraisals, context, and responses pertaining to any anomalous experiences endorsed from the inventory. It can also be used independently from the inventory to explore anomalies elicited with other clinical instruments (in this instance, the PSI). The format is flexible, and different sub-sections can be used to assess current anomalous experiences, lifetime anomalous experiences, and also changes in interpretation and response style over time. Assessing a person’s current style of appraising and responding takes approximately 10–15 min. The AANEX has been shown to reliably differentiate between clinical and non-clinical groups (24, 25).

### Procedure

Baseline data were collected from participants a few weeks prior to attending the testing facility (in order to minimize any anticipatory anxiety this may have caused on the day of the experiment itself). The baseline data-set for both groups included AANEX inventory state scores; STAI (full version); PSI. All participants submitted their data via an online website. In order to minimize order effects, participants in both groups were randomly split into two counterbalanced halves. The first half completed the deprivation condition first, followed by the seclusion condition separated by a half-hour break. These were reversed for the remainder. Following completion of the experiment, participants were debriefed, and received a nominal fee (the standard one set for psychology experiments) for their time in taking part.

### Deprivation condition

The anechoic chamber and associated procedure is described previously (17). The amendments were the absence of a panic-button and presence of a microphone so that participants could be heard externally by the experimenter should they become distressed. Participants were informed that if they wished to terminate the experiment at any point they should remain seated and tell the experimenter, who would immediately restore light and communication. No participants chose to terminate the experiment early. After a period of 25 min within the chamber, participants were moved to an ante-room where they were immediately asked to complete questionnaires referring to the time that they had spent in the anechoic chamber: the AANEX inventory (state items only); STAI (state items only); PSI. For participants who reported clear anomalous experiences, the AANEX-CAR interview was also administered to gather data on appraisal and responding styles.

### Secluded office condition

Participants were seated in an unoccupied office for same period of time as the sensory deprivation condition. They then completed the same questionnaires/interview: AANEX inventory (state items only); STAI (state items only); PSI. Once again, if participants reported clear anomalous experiences, the AANEX-CAR interview was administered to gather data on appraisal and responding styles.

## Results

### Preliminary statistical analyses

All statistical analyses were conducted using SPSS 17.0. Data were checked for normality before analysis using descriptive statistics and histograms with normal distribution curves. Anxiety and PSI scores were normally distributed; however, AANEX scores violated parametric assumptions due to significant floor effects, and as a result were not submitted to analysis of variance. Since the AANEX and PSI were both used to measure the underlying construct of PLEs, a non-parametric test of correlation (Kendall’s tau) was carried out to detect the strength of association between the two measures. There was a strong positive relationship between AANEX and PSI scores across all three conditions: baseline, τ = 0.54, *p* < 0.001; seclusion, τ = 0.70, *p* < 0.001; and deprivation, τ = 0.64, *p* < 0.001. This supported the validity of using PSI scores as the measure of PLEs in the main analysis, despite this measure not having been formally validated for use in this experimental context. Age and sex were unrelated to PSI and anxiety scores and so were not considered further in analysis.

The order in which participants experienced seclusion and deprivation conditions was counterbalanced as part of the experimental procedure, however, a preliminary mixed between-within subjects repeated measures analysis of variance was carried out to test for any effect of order on anxiety or PSI scores. A significant main effect of order was found for both anxiety scores [*F*(1,32) = 7.41, *p* < 0.01] and PSI scores [*F*(1,32) = 5.07, *p* < 0.05], with participants who experienced seclusion first reporting higher anxiety and PSI scores throughout the experiment. There were no interactions between order and group, indicating that these order effects are not dependent on degree of hallucination proneness.

### Baseline group comparisons

It was hypothesized that the high scoring group would score significantly higher on measures of psychotic-like symptoms under normal baseline conditions. The high and low scoring groups did differ significantly in PSI scores at baseline [*F*(1,34) = 6.145, *p* < 0.001], with the high scoring group reporting a greater number of psychosis-like experiences (see Table 1 for descriptives).

**Table 1:** **Questionnaire mean scores for high and low hallucination-prone groups by condition**.

Questionnaire scores	High scorers (*n* = 18)			Low scorers (*n* = 18)		

Revised hallucinations scale	54.94			26.22		

	Baseline	Seclusion	Deprivation	Baseline	Seclusion	Deprivation
Trait anxiety	47.78	–	–	31.89	–	–
State anxiety	42.78	36.33	38.89	32.50	33.17	36.17
AANEX	39.94	39.28	44.67	29.33	29.33	31.17
Psychotomimetic states inventory (sub-scales below)	37.00	36.83	49.28	13.83	19.89	27.11
Delusory thinking	4.83	4.94	5.50	2.17	1.78	2.22
Perceptual distortions	3.33	5.78	10.78	1.17	2.06	4.89
Cognitive disorganization	9.94	8.78	11.78	3.33	4.56	5.72
Anhedonia	9.17	8.06	10.56	3.67	6.83	8.72
Mania	5.89	6.17	7.28	2.78	3.89	4.50
Paranoia	3.83	3.11	3.39	0.72	0.78	1.06

Baseline trait and state anxiety scores were significantly correlated (*r* = 0.74, *p* < 0.001). Significant differences in trait anxiety [*F*(1,34) = 20.23, *p* < 0.001] and state anxiety [*F*(1,34) = 7.91, *p* < 0.01] were found between the high and low hallucination prone groups at baseline, with the high hallucination prone group reporting higher levels of trait anxiety (*x* = 47.78, SD = 12.95 compared to 31.89, SD = 7.55) and state anxiety (*x* = 42.28, SD = 11.83 compared to 32.50, SE = 8.82). Although not specifically hypothesized all the above findings are in the expected direction.

### Psychosis-like experiences across groups and conditions

**Figure 1:**
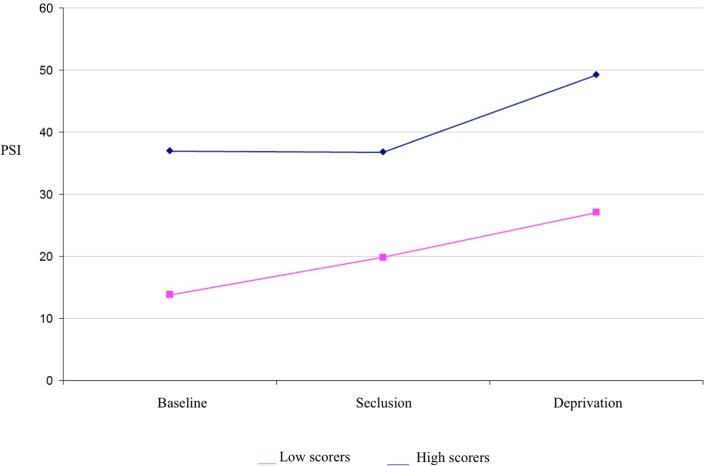
**Psychotomimetic states inventory scores in high and low hallucination-prone groups by condition**.

It was hypothesized that while both groups would experience a significant increase in psychosis-like symptoms from baseline in near-total sensory deprivation, the increase would be significantly greater for the high scoring group. Results of a mixed between-within subjects repeated measures analysis of variance demonstrated a significant main effect of group for PSI scores [*F*(1,34) = 31.31, *p* < 0.001] (see Table 1 for descriptives). This indicates that the high hallucination prone group experienced a significantly greater number of psychosis-like symptoms overall throughout the experiment, independent of condition (see Figure 1).

There was also a main effect of condition for PSI scores [*F*(1,83) = 12.524, *p* < 0.001] (see Table 1 for descriptives). Planned contrasts revealed that PSI scores were significantly higher in deprivation than at baseline [*F*(1,34) = 17.86, *p* < 0.001] and PSI scores were significantly higher in deprivation than in seclusion [*F*(1,34) = 14.05, *p* < 0.001]. There was no significant difference in PSI scores between seclusion and baseline. There was no interaction effect detected between group and condition, suggesting that both high and low scoring groups responded in a similar way to the experimental conditions.

A further mixed between-within subjects repeated measures analysis of variance examining the PSI sub-scales of delusional thinking, perceptual distortion, cognitive disorganization, anhedonia, mania, and paranoia was conducted to investigate any difference in particular types of psychosis-like experiences reported across the different conditions. Consistent with hypotheses, there was a significant main effect of condition for perceptual distortions [*F*(2,68) = 34.15, *p* < 0.001], anhedonia [*F*(2,68) = 10.76, *p* < 0.001], mania [*F*(2,68) = 6.53, *p* < 0.01], and cognitive disorganization [*F*(2,68) = 3.22, *p* < 0.05]. Planned contrasts indicated that perceptual distortions and anhedonia scores were significantly higher in seclusion than at baseline, and further increased during deprivation. Mania and cognitive disorganization were also significantly higher during deprivation than at baseline, but did not increase significantly in seclusion (see Table 1). A significant interaction between group and condition was found for the perceptual distortions subscale [*F*(2,68) = 3.63, *p* < 0.05], with high scorers showing a greater increase in these symptoms in deprivation than low scorers (a difference of around two SD, see Table 1). A significant interaction between group and condition was also found for the anhedonia subscale [*F*(2,68) = 5.31, *p* < 0.01], with low scorers showing a more marked increase in anhedonic symptoms in deprivation than high scorers (see Table 1).

### State and trait anxiety across groups and conditions

**Figure 2:**
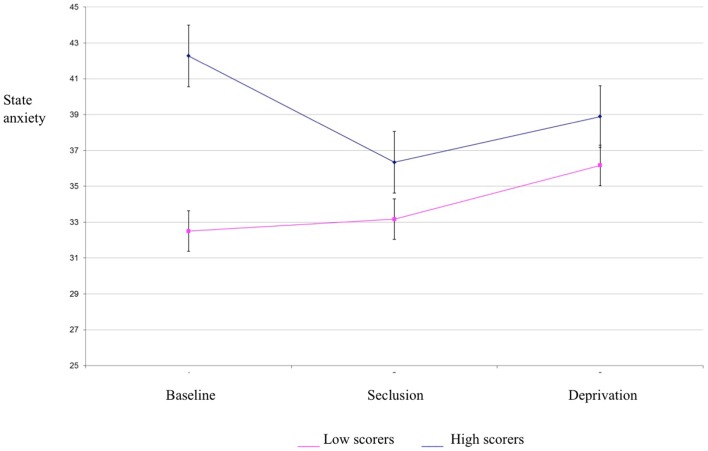
**State anxiety scores in high and low hallucination-prone groups by condition**.

Results of a mixed between-within subjects repeated measures analysis of variance demonstrated a significant main effect of group for state anxiety scores [*F*(1,34) = 4.21, *p* < 0.05] (see Table 1 for descriptives). This indicates that the high hallucination prone group experienced higher state anxiety than the low hallucination prone group. There was no effect of condition for state anxiety, suggesting that anxiety did not differ between baseline, seclusion, and deprivation conditions (see Figure 2). Thus, state anxiety is unlikely to account for the differences in psychosis-like experiences between conditions. Trait anxiety differed between experimental groups, but did not correlate with PSI scores in any condition. Consequently, trait anxiety was not considered as a covariate for analysis of variance for PSI scores.

### AANEX-CAR

AANEX-CAR semi-structured interviews were administered to all participants who reported clearly identifiable psychosis-like experiences in seclusion or deprivation. Interviews were indicated for 11 out of 18 participants in the high scoring group, and 4 out of 18 participants in the low scoring group. Consistent with PSI results, the hallucination prone group reported a greater number of psychosis-like experiences than the non-prone group (χ^2^ = 4.11, *p* < 0.005).

All interviews were indicated following experiences in deprivation. The types of experiences participants reported were varied, including hearing noises such as insects buzzing and whistling (*n* = 2); hearing music (*n* = 2); seeing shapes and colored lights (*n* = 4); visual hallucinations such as seeing faces and animals (*n* = 2); out-of-body experiences or the experience of watching events through another’s eyes (*n* = 3); disorientation such as feelings of falling, the room spinning, and the walls closing in (*n* = 2). Interviews were scored according to the procedure described by Brett et al. (24), and ratings derived for appraisal dimensions, appraisal categories, emotional response, cognitive and behavioral response, perceived social understanding, and perceived controllability.

In order to establish whether AANEX-CAR scores reflected typical appraisal and cognitive/emotional response styles of people experiencing genuine symptoms that had not been experimentally “induced,” the scores were compared with existing data from a clinical group (schizophrenia spectrum disorders) and a non-clinical group with anomalous experiences [Ref. (25), see Table 2]. Experiences under sensory deprivation were similar to those seen in the non-clinical group and differed from the clinical group in being appraised as less dangerous, less external, less due to others, and less anxiety provoking/negative emotionally; and as having a greater sense of agency, and more likely to have a psychological cause. However, the sensory deprivation group’s appraisals differed from the non-clinical group in not being as positively valenced; not as spiritual in meaning; or positive emotionally. In these latter respects they did not differ significantly from the clinical group.

**Table 2:** **Appraisals under sensory deprivation compared with Lovatt et al. groups (25)**.

AANEX-CAR items	Sensory deprivation group (*n* = 15) mean (SD)	Clinical group (*n* = 29) mean (SD)	Non-clinical group (*n* = 29) mean (SD)	*F* test	*Post hoc* comparisons (Scheffe)
**APPRAISAL: DIMENSIONS**					
Valence	2.93 (1.24)	2.52 (1.25)	4.19 (1.04)	14.23**	NC > C = SD
Dangerousness	2.66 (1.74)	3.81 (1.18)	2.74 (1.10)	5.85**	C > NC = SD
Externality	2.00 (1.10)	3.44 (1.25)	2.33 (0.92)	10.61**	C > NC = SD
Agency	4.47 (0.72)	3.85 (1.2)	2.44 (1.15)	19.46**	C > NC = SD
**APPRAISAL: CATEGORIES**					
Biological	0.07 (0.25)	0.48 (0.80)	0.44 (0.80)	n.s	–
Psychological/normalizing	2.00 (0.00)	0.44 (0.75)	1.44 (0.75)	29.90**	C > NC = SD
Spiritual	0.33 (0.70)	0.67 (0.78)	1.33 (0.88)	8.54**	NC > C = SD
Other people	0.07 (0.25)	1.11 (0.93)	0.74 (0.26)	23.03**	C > NC = SD
**EMOTIONAL RESPONSE**					
Neutral arousal	2.33 (1.19)	2.59 (1.25)	2.70 (1.07)	n.s	–
Negative emotional response	2.12 (1.54)	3.70 (1.07)	2.00 (0.92)	17.09**	C > NC = SD
Positive emotional response	2.07 (1.24)	2.19 (0.92)	3.15 (1.13)	7.05**	NC > C = SD
Self-rated anxiety	2.53 (1.31)	3.96 (1.02)	1.96 (1.06)	22.69**	C > NC = SD
Self-rated excitement	2.27 (1.34)	2.48 (1.48)	3.03 (1.45)	n.s.	–

*NC, non-clinical group; C, clinical group; SD, sensory deprivation group*.

**p < 0.01.

## Discussion

Consistent with hypotheses, hallucination proneness was associated with greater psychosis-like symptoms under all conditions. In addition, both high and low scoring groups experienced a significant increase in psychosis-like symptoms in sensory deprivation conditions. Sensory deprivation was found to produce a significant increase on four sub-scales of the PSI: perceptual distortions, anhedonia, mania, and cognitive disorganization. Findings with respect to perceptual distortions and anhedonia were highly marked and were consistent with the pilot study (17). However, unlike the previous study, paranoia did not appear to increase significantly. As the current study is larger and so better powered, and utilized a longer time period, it is likely to provide a more sensitive profile of the psychotic-like symptoms provoked by deprivation. In the current study, an interaction effect between condition and group was only seen for the perceptual distortion subscale clearly validating the RHS and suggesting a state-trait interaction. Consistent with this, the majority of hallucination prone individuals (11 of 19) reported clear anomalous experiences sufficient for AANEX-CAR interview, in contrast with a minority of non-prone (4 of 19). The potential role of state and trait anxiety was explored. Consistent with the previous literature (21), trait and state anxiety distinguished the high hallucination-prone from the low hallucination-prone groups at baseline. However, trait anxiety neither predicted changes in PSI scores nor differed across condition in either group. Therefore, the increase in psychosis-like symptoms seen in both groups during deprivation cannot be readily attributed to increased anxiety.

The considerable presence of anomalous perceptions that were experienced to some degree at least as autonomous, external, and “hallucination-like” are consistent with the “faulty source monitoring” hypothesis (9). It is also consistent with the framework offered by Fletcher and Frith (13) that unusual perceptions arise out of an abnormality in the brains’ inferencing mechanism, so that new evidence (including sensations) is not properly integrated, leading to false prediction errors in psychotic and psychosis-prone individuals. In the absence of external stimuli, perceptual distortions are presumably internally generated by the individuals, but are misattributed as external in origin due to “top-down” processes (12). Overall, the range and frequency of psychotic-like symptoms are sufficient to endorse Corlett et al.’s (12) position that sensory deprivation offers a promising model of psychosis in psychiatrically healthy individuals. Future research should explore the underlying neurocognitive mechanisms of PLEs under sensory deprivation using neurobiological methods such as psychophysiological recording.

Also of interest, but not predicted, was that *low* scorers experienced a significantly greater increase in anhedonic symptoms during deprivation as compared to baseline measurement. Previously, this finding had only been seen in high scorers. This could be due to boredom effects in the low scoring group (related to the longer time duration), who otherwise reported few psychosis-like experiences during deprivation.

AANEX-CAR data showed the appraisal and cognitive/emotional response styles of participants were broadly consistent with those of non-clinical individuals with anomalous experiences. Participants strongly believed that the causes of their experiences were psychological in nature and that they had some agency within them. The unusual environmental context may have made them more likely to interpret their experiences in terms of internal mental processes. Anxiety, dangerousness, and a negative emotional response were at the low levels seen in non-clinical individuals, and unlike the symptomatic experiences of those with psychotic disorders. However, non-clinical individuals with repeated anomalous experiences have often been shown to develop positively valenced appraisals with, for some, strong spiritual meanings. This did not prove the case, in general, for those in sensory deprivation. “Naturally” occurring – and reoccurring – anomalous experiences are plausibly more likely to develop idiosyncratic and personally highly meaningful appraisals than those “artificially” created by laboratory conditions.

### Limitations

Despite attempting to address several potential confounds of the pilot study, other such as social desirability are suggestibility cannot be excluded and deserve further testing. Though the appraisal data go some way to detailing the similarities with clinical and non-clinical psychotic experiences there is some way to go before concluding the phenomena seen in sensory deprivation are equivalent as this is currently reliant on self-report. Biometric approaches such as psychophysiological or neurocognitive indices would clearly strengthen the argument.

The “secluded office” condition attempted to provide a closer analog to sensory deprivation (in duration at least) than the baseline but this was not highly successful. While, on many indices these two conditions appeared highly similar there were significant order effects across both groups, with participants who experienced seclusion first reporting more psychosis-like experiences throughout the experiment. It is possible that participants who experienced seclusion first responded to the perceived demand characteristics of the experiment, endorsing more items on the PSI and AANEX measures in this first condition. Counter-balancing was incorporated into the experimental design in an attempt to moderate any order effects, but demand characteristics may still have had some impact particularly on the seclusion data. As a consequence, the baseline condition is very probably the more stable one against which to compare the experimental deprivation condition.

### Clinical implications

The findings suggest that even during a quite brief period of sensory deprivation, perceptual distortions, and other psychosis-like experiences are common in the “normal” population. Although high hallucination prone individuals reported significantly greater levels of perceptual distortions, individuals not prone to experiencing hallucinations were also affected. In the current study, any psychosis-like symptoms were transient, and quickly resolved once participants were returned to normal conditions. Indeed, the distortions and other psychotic phenomena induced did not bring attendant anxiety as probably occurs with many early psychotic symptoms. Nevertheless, it is possible that a longer period of deprivation may have the potential to induce enduring symptoms of psychosis with consequent distress.

## Conclusion

Overall, the study provides further support for use of sensory deprivation as a non-pharmacological tool for temporarily inducing psychotic-like states. Both high and low hallucination prone groups responded to sensory deprivation in a qualitatively similar manner, but with quantitative differences in the frequency of psychosis-like experiences reported. It appears possible to accurately predict individuals who are most likely to experience psychosis-like experiences in sensory deprivation based on the presence/absence of schizotypal traits (here as indexed by hallucination proneness). Sensory deprivation would seem a useful paradigm to model psychotic symptoms, to which we would add the important ethical principle of non-harm.

## Conflict of Interest Statement

The authors declare that the research was conducted in the absence of any commercial or financial relationships that could be construed as a potential conflict of interest.
